# Successful Repair of a Complete Tracheobronchial Tear Two Months After the Injury

**DOI:** 10.7759/cureus.31628

**Published:** 2022-11-18

**Authors:** Imran Tahir, Qaidar Alizai, Farhan Ullah, Abdul Haseeb, Nadeem Ijaz

**Affiliations:** 1 Department of Surgery, Hayatabad Medical Complex Peshawar, Peshawar, PAK; 2 Department of Internal Medicine, Khyber Teaching Hospital MTI, Peshawar, PAK; 3 Department of Surgery, Khyber Teaching Hospital MTI, Peshawar, PAK

**Keywords:** video bronchoscopy, postero-lateral thoracotomy, blunt chest injuries, chest computed tomogram, tracheobronchial injury

## Abstract

Tracheobronchial injury (TBI) is a rare but potentially life-threatening tear of the lower airway that can result from iatrogenic or accidental trauma. We present a case of a young male who suffered from acute TBI following blunt trauma to the chest. The patient was managed conservatively with intubation and oxygen support initially. The condition improved and the patient was discharged. However, he developed chest pain two months later and was diagnosed with a complete TBI on the right side. He subsequently underwent open surgical repair of the tear with end-to-end anastomosis, which led to a full recovery.

## Introduction

Traumatic airway injury (TAI) is a rare but life-threatening condition and accounts for less than 1% of all trauma patients in most studies [1]. Tracheobronchial injury (TBI) is a subtype of airway injury that involves the airway anywhere from the level of the cricoid cartilage up to the bronchial tree. It may be iatrogenic or can be caused by blunt or penetrating trauma [2]. Iatrogenic injuries are more common in the posterior membranous part of the trachea. In penetrating trauma and overall, cervical tracheal injury is more common. However, in blunt trauma, most of the injuries are intrathoracic [3].

About 80% of the TBI cases associated with blunt trauma die before reaching the hospital due to severe associated injuries, and the involvement of vital organs and major vessels of the neck and chest. Survival depends on a timely pre-hospital rescue, a high degree of suspicion, early diagnosis, and immediate management. The initial management starts with resuscitation, a chest X-ray (CXR), and an X-ray of the neck. A CT of the chest can confirm the diagnosis in equivocal cases [2]. However, flexible fiberoptic bronchoscopy remains the diagnostic modality of choice regardless of the CT findings [4,5]. While minor injuries may be managed with conservative treatment alone, severe cases will require open or thoracoscopic surgical repair [3].

In rare cases, the patients' condition will improve after the initial conservative management itself, only for them to return with pulmonary signs and symptoms months to years later. In this report, we present a rare case of complete TBI after a blunt chest trauma that was repaired successfully two months after the initial insult, without any resection of the lung tissue. This case strengthens the evidence for successful late surgical repair of significant TBI without any viable lung tissue loss.

## Case presentation

A 26-year-old South Asian construction worker sustained a blunt chest trauma following a low-velocity road traffic accident (pedestrian vs. excavator machine). He was taken to the hospital, anxious, and in respiratory distress. An emergency CXR confirmed bilateral hemopneumothorax for which chest tubes were placed and his symptoms began to improve, but he was still in respiratory distress. He was admitted to the ICU and put on oxygen support via a venturi face mask to maintain oxygen saturation above 94%. As his condition improved, the left chest tube was removed first on day six. The right chest tube was removed at discharge, approximately 14 days after admission.

**Figure 1:**
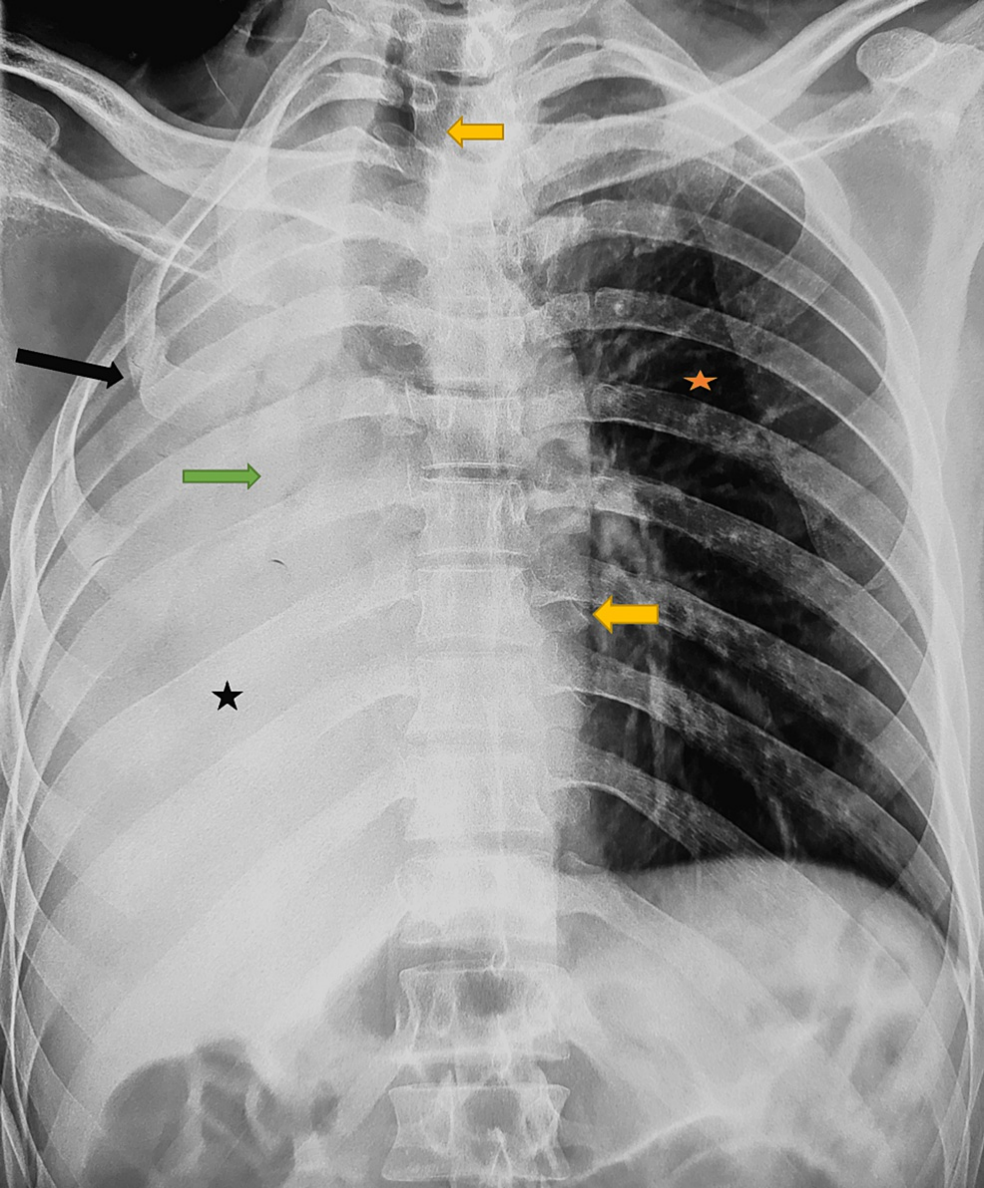
Preoperative chest X-ray, posteroanterior view The image shows diffuse white-out of the right hemithorax (black star), and abrupt cutoff of the right bronchus (green arrow) along with ipsilateral mediastinal and tracheal shift (yellow arrows). The fractured right second rib (black arrow) can also be seen. The left lung appears normal but has compensatory over-expansion (orange star)

Unfortunately, six weeks after his discharge, the patient developed progressive right-sided chest pain and gradually increasing shortness of breath. There was no history of any new trauma. Repeat CXR revealed moderate right hemothorax and a collapsed right lung, along with a mediastinal and tracheal shift to the same side (Figure 1). This was suggestive of a blocked or disrupted right-sided airway with secondary atelectasis. Hence, he was referred to the thoracic surgery unit.

**Figure 2:**
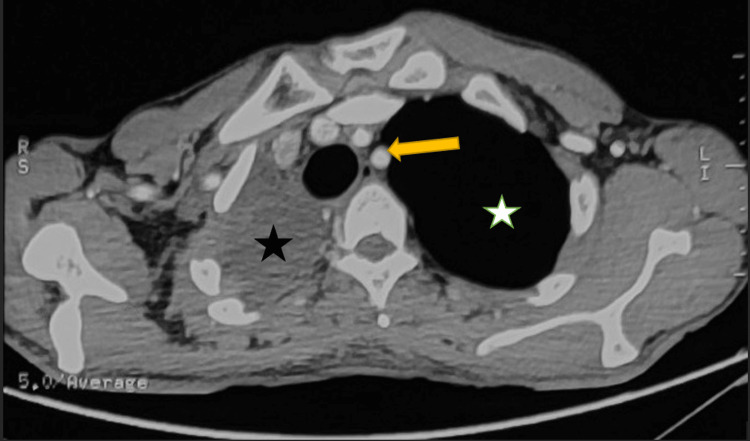
CT chest without contrast (transverse section) at the level of the upper thorax The image shows a moderate right-sided hemothorax (black star) along with a mediastinal and tracheal shift (yellow arrow) to the same side. There is compensatory hyper-expansion of the left lung (white star) CT: computed tomography

**Figure 3:**
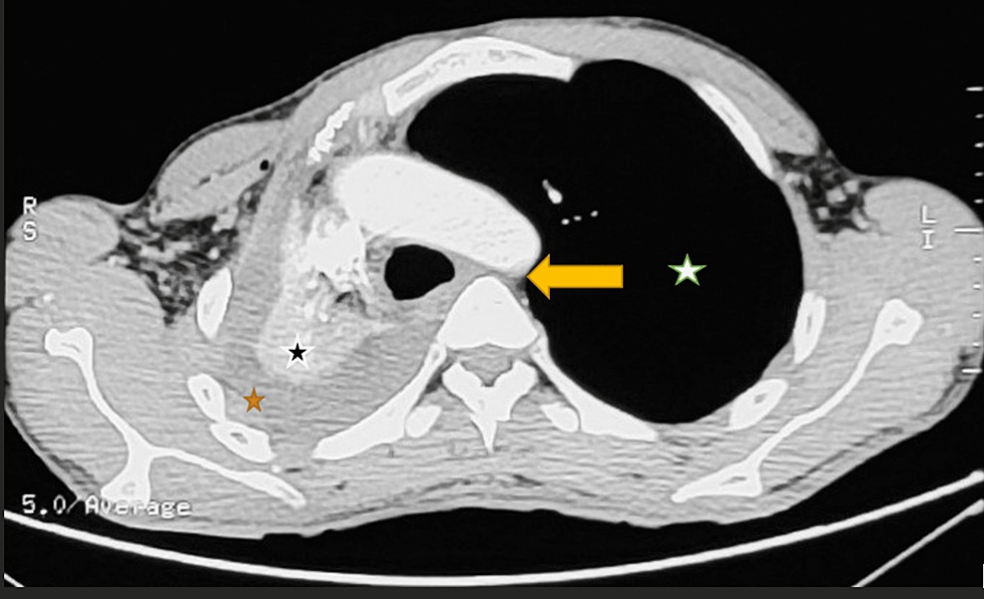
CT chest without contrast (transverse section) at the level of the mid thorax The image shows a collapsed right lung (orange star) along with moderate hemothorax (black star). It also shows a mediastinal shift and tracheal shift (yellow arrow) to the right and a compensatory over-expansion of the left lung (white star) CT: computed tomography

**Figure 4:**
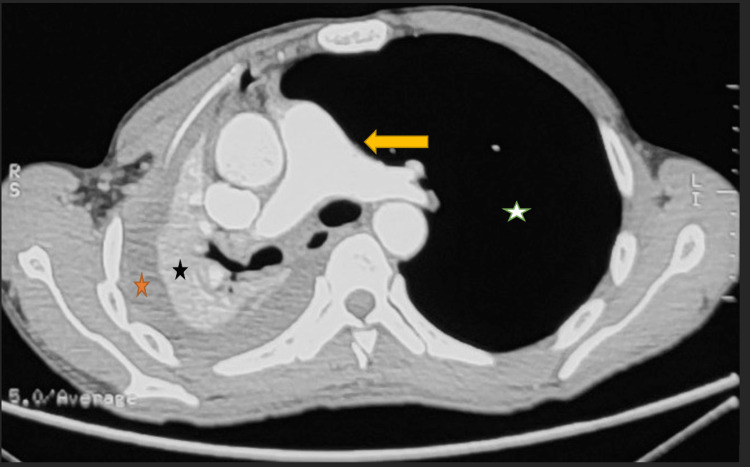
CT chest without contrast (transverse section) at the level of the lower thorax The image shows a collapsed lung (black star), a moderate right-sided hemothorax (orange star) along with a mediastinal shift to the same side (yellow arrow). There is a compensatory expansion of the left lung (white star) CT: computed tomography

On arrival, the patient was conscious and well-oriented but in mild respiratory distress. His oxygen saturation was 85% on room air. On examination, breath sounds were absent on the right side of the chest with dull percussion on the same side. The CT chest revealed gross right-sided hemothorax and complete right lung collapse with an air bronchogram. There was a resultant ipsilateral mediastinal shift and over-expansion of the left lung suggestive of a complete TBI on the right (Figures 2-4). Fiberoptic bronchoscopy was not done due to the unavailability of resources. Due to a high suspicion of a complete TBI, the decision was made to perform an open surgical repair.

After providing informed consent, the patient underwent a right posterolateral thoracotomy. A complete disruption of the right main bronchus was found 1 cm below the carina, with a collapse of the distal lung. The inferior pulmonary ligament was released and the right main bronchus was mobilized above and below the injury. Edges of the bronchus were refreshed, and the bronchus was repaired with end-to-end anastomosis with a non-absorbable Prolene 1 suture. An intercostal muscle flap was applied for reinforcement. Two chest drains were placed in the right pleural cavity. After securing hemostasis, the wound was closed in reverse order and an antiseptic dressing was applied. He had an uneventful post-anesthesia recovery and was shifted to the ward with close monitoring.

**Figure 5:**
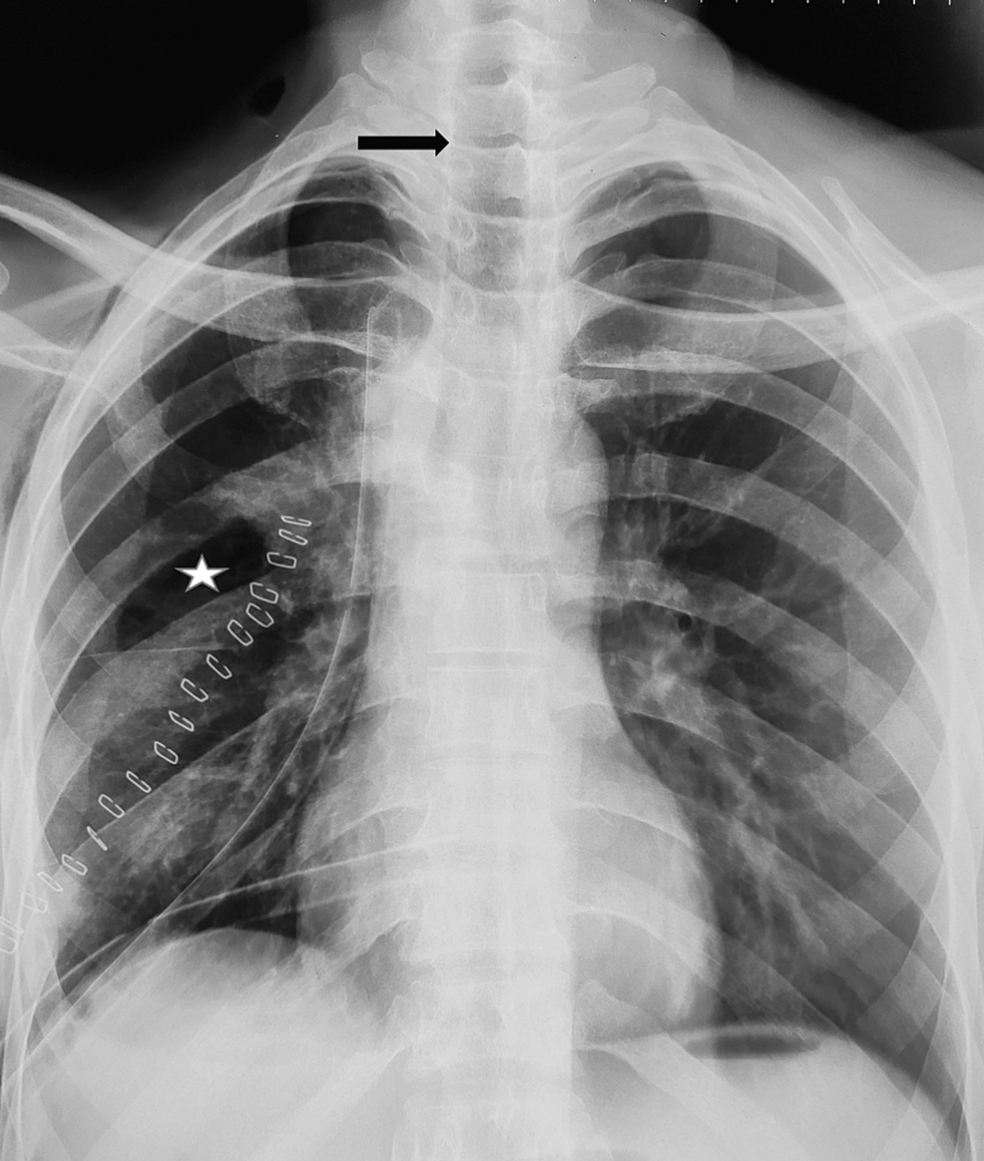
Postoperative chest X-ray showing an expanded right lung (star) with midline trachea (arrow), consistent with a successful bronchial anastomosis

The patient's condition improved both clinically and radiologically after surgery. A follow-up CXR revealed a fully expanded right lung and midline mediastinum (Figure 5). A postoperative video bronchoscopy confirmed the successful repair of the injury with a healthy anastomosis. He was kept under observation and later discharged on the third postoperative day after the removal of one chest drain. At the two-week follow-up, the patient had significant improvement, and hence the second drain was also removed. The patient remained healthy on follow-up at six months.

## Discussion

TBI is a rare form of airway injury in trauma patients. In penetrating trauma and in general, cervical tracheal injury is more common than TBI. However, blunt trauma is commonly associated with intrathoracic TBI [3,5]. Most of the injuries occur near the carina. According to one study, injuries were located within 2 cm of the carina in up to 76% of TBI patients. Among the two bronchi, the right main bronchus is more prone to tears due to multiple reasons: the heavier mass of the right lung and the shorter length of the right bronchus make it prone to traction injury. In addition, the right bronchus is less protected by the aorta and other mediastinal organs unlike the left bronchus [6]. Moreover, TBI from blunt trauma results in transverse tears in 74% of cases, and about 33% of such injuries have longitudinal tears [7]. Our patient had a complete transverse transection of the right bronchus 1 cm from the carina.

Based on the depth of the tear, the TBI can be classified into partial and complete tears. When the diagnosis of a partial TBI is missed initially, the patient may develop granulation tissue and stricture at the site of injury. This leads to infection, bronchiectasis, and even lung abscesses. Paradoxically, in a complete airway injury with obstruction, the distal lung will be filled with mucus and protected from infections. These patients will have intact parenchyma and a better prognosis for repair. A complete tear may be evident on the CT scan of the chest as a collapsed lung lying on the floor of the thoracic cavity, completely separated from the trachea. Furthermore, it may be a small tear (<4 cm longitudinally) or a larger tear (>4 cm). A small, partial tear can be treated nonoperatively with chest intubation. However, complete and/or larger tears will require open surgical repair, as in our case [5].

The treatment of TBI depends on multiple factors, including site, size, cause of injury, mechanism of injury, patient condition, and presentation. Most of the very small TBIs will heal spontaneously. Some of the tears will improve with chest intubation and oxygen support. Small upper tracheal tears can be managed with an endotracheal tube passed under fibroscopic visualization and its cuff inflated past the site of the tear. However, large tears will require open surgical repair [5]. Lobectomy may be indicated in some cases where the small bronchi are injured beyond repair. Poor surgical candidates may benefit from tracheal stent placement [7,8].

Most of the cases that survive the journey to the operating room will have a complete recovery after surgery. Repaired TBIs have a better outcome than those receiving no treatment at all or those that receive conservative treatment only. Furthermore, a bronchial repair has a better prognosis than surgical resection of the bronchus and the lung [6]. Finally, a late presenting complete airway disruption and/or obstruction, paradoxically, will have a better outcome due to preserved distal lung parenchyma [5]. These cases can be repaired without any resection of lung tissue. This was evident in our patient as well who underwent open surgical repair two months after the injury and recovered successfully.

## Conclusions

Based on this case report, it is evident that complete tracheobronchial tears can be successfully repaired even months after the initial injury without any loss of lung tissue. A suspected TBI that is refractory to conservative management should undergo surgical repair. We recommend that even a chronic complete TBI should be repaired without any resection unless there are other indications.
